# Gene‒environment interaction effect of hypothalamic‒pituitary‒adrenal axis gene polymorphisms and job stress on the risk of sleep disturbances

**DOI:** 10.7717/peerj.17119

**Published:** 2024-03-20

**Authors:** Min Zhao, Yuxi Wang, Yidan Zeng, Huimin Huang, Tong Xu, Baoying Liu, Chuancheng Wu, Xiufeng Luo, Yu Jiang

**Affiliations:** 1 Department of Public Health, Fujian Medical University, Fuzhou, China; 2 Affiliated Zhongshan Hospital of Dalian University, Dalian, China; 3 Fuzhou Municipal Center for Disease Control and Prevention, Fuzhou, China

**Keywords:** Job stress, Sleep disturbance, HPA axis, Gene‒environment interaction, Rail workers

## Abstract

**Background:**

Studies have shown that chronic exposure to job stress may increase the risk of sleep disturbances and that hypothalamic‒pituitary‒adrenal (HPA) axis gene polymorphisms may play an important role in the psychopathologic mechanisms of sleep disturbances. However, the interactions among job stress, gene polymorphisms and sleep disturbances have not been examined from the perspective of the HPA axis. This study aimed to know whether job stress is a risk factor for sleep disturbances and to further explore the effect of the HPA axis gene × job stress interaction on sleep disturbances among railway workers.

**Methods:**

In this cross-sectional study, 671 participants (363 males and 308 females) from the China Railway Fuzhou Branch were included. Sleep disturbances were evaluated with the Pittsburgh Sleep Quality Index (PSQI), and job stress was measured with the Effort-Reward Imbalance scale (ERI). Generalized multivariate dimensionality reduction (GMDR) models were used to assess gene‒environment interactions.

**Results:**

We found a significant positive correlation between job stress and sleep disturbances (*P* < 0.01). The *FKBP5* rs1360780-T and rs4713916-A alleles and the *CRHR1* rs110402-G allele were associated with increased sleep disturbance risk, with adjusted ORs (95% CIs) of 1.75 [1.38–2.22], 1.68 [1.30–2.18] and 1.43 [1.09–1.87], respectively. However, the *FKBP5* rs9470080-T allele was a protective factor against sleep disturbances, with an OR (95% CI) of 0.65 [0.51–0.83]. GMDR analysis indicated that under job stress, individuals with the *FKBP5* rs1368780-CT, rs4713916-GG, and rs9470080-CT genotypes and the *CRHR1* rs110402-AA genotype had the greatest risk of sleep disturbances.

**Conclusions:**

Individuals carrying risk alleles who experience job stress may be at increased risk of sleep disturbances. These findings may provide new insights into stress-related sleep disturbances in occupational populations.

## Introduction

Sleep is essential for humans, helping to maintain energy, promote growth and development, and improve immunity (Ramar et al., 2021). However, sleep disturbances seriously reduce people’s quality of life and have become a major public health problem affecting people’s physical and mental health (Halonen et al., 2017). The global prevalence of sleep disturbances is approximately 37.9% (Wu et al., 2021); in Canada, the prevalence is 23.8% (Chaput et al., 2018); in Japan, the prevalence is 13.3% (Miyachi et al., 2021); in the US, the prevalence is 30.5% (Kadier et al., 2023); in Europe, the prevalence is 25.73% (Linh et al., 2023); in Africa, the prevalence is 32.6% (Wang et al., 2019); and in China, the prevalence is 29.2% (Shi et al., 2020). Long-term sleep disturbances negatively affect people’s physical and mental health and are early risk factors for many diseases, such as cardiovascular and cerebrovascular diseases, neuropsychiatric disorders, accidental injuries and even death (Rajaratnam et al., 2011; Morin & Jarrin, 2022).

Job stress is a negative physical and psychological reaction that occurs when job requirements do not match workers’ abilities, coping resources and demands (Basu, Qayyum & Mason, 2017). In recent years, studies have shown that excessive job stress can lead to imbalances in physiological functions, resulting in decreased sleep quality and sleep problems such as insomnia and drowsiness (Khamisa et al., 2016; Herr et al., 2018; D’Ettorre et al., 2020). Therefore, job-related stress is a major occupational risk factor that significantly increases the risk of sleep disturbances (Juster & McEwen, 2015; Linton et al., 2015). Epidemiological research has indicated that job stress is related to an increased risk of sleep disturbances (Blom et al., 2020; Hämmig, 2020). A prospective cohort study of Japanese workers (*n* = 1,022) with a 2-year observation period also revealed that high job stress was strongly associated with insomnia, with an OR (95% CI) of 1.72 [1.06–2.79] (Ota et al., 2009). A prospective cohort study of workers in Denmark aligns with this conclusion (Nordentoft et al., 2020). In addition, sleep disturbances also seriously affect the efficiency of workers, leading to a decline in production efficiency and the occurrence of accidents, resulting in substantial social and economic burdens (Kucharczyk, Morgan & Hall, 2012; Uehli et al., 2014). It is essential to explore the mechanism underlying the influence of job stress on sleep disturbances among occupational groups and to take active measures to reduce the occurrence of sleep disturbances.

The HPA axis is thought to be the main pathway mediating the stress response (Hirotsu, Tufik & Andersen, 2015). More importantly, the HPA axis regulates the sleep-wake cycle: activation of the HPA axis may lead to awakening and insomnia in animals and humans (de Feijter et al., 2022). Dysfunction of the HPA axis at any molecule (such as the corticotropin-releasing hormone receptor, glucocorticoid receptor or mineralocorticoid receptor) may disturb sleep (Buckley & Schatzberg, 2005). When encountering stressors (physiological or psychological), the hypothalamus releases corticotropin-releasing hormone (CRH). CRH stimulates the anterior pituitary to release corticotropin, and corticotropin activates the adrenal cortex to upregulate the production of glucocorticoids (GCs). Its main function is to restore internal physiological balance after exposure to stress. However, Weitzman et al. (1983) showed that the release of GCs was related to the occurrence and development of sleep disturbances. Moreover, most stress-related hormones promote wakefulness, and elevated HPA activity appears to contribute to stress-induced insomnia (Nicolaides, Vgontzas & Kritikou, 2000). Exploring the genes that play a role in HPA axis regulation may be useful in determining the relationship between job stress and sleep disturbances. Gerritsen et al. (2017) suggested that the *CRH* gene is linked to stress and sleep disturbances. In addition, individual variation in the FK506 binding protein five (*FKBP5*) gene is related to an imbalance in the HPA axis; this imbalance has been identified as the key neurobiological mechanism underlying psychotic symptoms (Nold et al., 2022; Wang et al., 2023). An animal study also reported that *FKBP5* may be a target gene for stress-induced mood and sleep disturbances (Albu et al., 2014). Although many studies have shown that sleep disturbances are related to HPA axis genes and job stress, their interaction and effect on sleep disturbances remain unclear.

In recent years, many researchers have assessed the effects of gene‒environment interactions on sleep disturbances (Zwicker, Denovan-Wright & Uher, 2018; Zhang, Khan & Rzhetsky, 2022). Both genetic (Federenko et al., 2004) and environmental factors have been shown to influence an individual’s cortisol response to stress through the HPA axis, even if the response is extreme enough to increase the risk of sleep disturbances (Foley & Kirschbaum, 2010; Kudielka & Wüst, 2010). Moreover, interactions between several genes (the glucocorticoid receptor (*GR))* (Bakker et al., 2017), *FKBP5* (Matosin, Halldorsdottir & Binder, 2018; Normann & Buttenschøn, 2020), 5-hydroxytryptamine transporter (*5-HTTLPR)* (Huang et al., 2014) and dopamine D2 receptor (*DRD2)* (Jiang et al., 2020) and exposure to job stress have repeatedly been found to play a role in the onset of sleep disturbances. For instance, Brummett et al. (2007) reported that the *5-HTTLPR* gene polymorphism is related to sleep quality problems in individuals exposed to long-term stress. A previous study reported that the effects of early-life stress on mental illnesses such as sleep disturbances were more prominent for the G alleles of the *GR* genes rs258747 and rs41423247 (Lian et al., 2014). One of the largest Trier Social Stress Test (TSST) cohorts indicated that interactions among *FKBP5*, corticotrophin-releasing hormone receptor type 1 gene (*CRHR1*) gene polymorphisms and psychosocial stress may affect the cortisol response and cause circadian rhythm disruption (Mahon et al., 2013). However, there are still single nucleotide polymorphisms (SNPs) in the HPA axis that have not been fully investigated in these interactions. Most studies have focused on the effect of a single gene-stress interaction on sleep quality, and few have examined multiple major genes regulating the HPA axis to determine the relationships among gene polymorphisms, job stress, and their interaction with sleep disturbances.

Therefore, we chose SNPs of several major genes regulating the HPA axis to investigate the independent and interactive effects of HPA axis gene polymorphisms and job stress on sleep quality among front-line railway workers in Fuzhou city, China. Our investigation focused on the interaction effect of genetic and environmental factors on sleep disturbances to provide new insights for improving sleep health.

## Materials and Methods

### Subjects

The present study was conducted as part of the Occupational Health Study for Railway Workers (OHSRW) between October 2019 and May 2020. The inclusion and exclusion criteria have been described in detail in a previous article (Wang et al., 2022b). A set of self-report questionnaires was used to collect information on demographic characteristics, sleep disturbances and job stress. As a part of the physical examination, 5-mL fasting venous blood samples were collected from each subject at the workplace between 7:00 am and 9:00 am. In this cross-sectional study, a total of 690 participants were enrolled, 19 of whom were excluded due to insufficient information or missing blood samples. Ultimately, 671 (males/females = 363/308) railway front-line workers were included in the final analysis. This study was approved by the Ethics Committee of Fujian Medical University (No. 2019025). All subjects provided informed consent before they participated in the study and signed a written informed consent form.

### Job stress

The Effort-Reward Imbalance (ERI) scale, which is based on Siegrist’s ERI model, was used to evaluate job stress (Siegrist & Li, 2017). The Cronbach’s alpha of this scale was 0.882. The ERI questionnaire includes a total of 23 items in three dimensions: job effort (six items), job reward (six items) and overcommitment (11 items). Each of the items is evaluated on a five-point scale (from 1 to 5). The ERI score evaluation method is as follows: each item, is assigned the same weight, and the ERI score is calculated as E/(R × (6/11)). ERI scores >1 indicate an imbalance between effort and reward, which is considered to reflect job stress (Choi et al., 2014).

### Sleep disturbances

The Pittsburgh Sleep Quality Index (PSQI) was used to assess the sleep quality of the subjects (Buysse et al., 1989). The PSQI has shown strong reliability and validity in a variety of samples, indicating that this questionnaire provides a good understanding of sleep disturbances (Mollayeva et al., 2016). The PSQI consists of seven components: subjective sleep quality, sleep latency, sleep duration, sleep efficiency, sleep disturbance, sleep medication, and daytime dysfunction. Each dimension is graded on a scale ranging from 0 to 3, and the total PSQI score ranges from 0 to 21. The higher the score is, the worse the sleep quality. It has been reported that the PSQI is an easily accepted and applied tool for assessing sleep disturbances, with a score of ≥5 indicating that the subject has significantly poorer sleep quality. In this study, subjects with a global score higher than five were classified as experiencing sleep disturbances (Yilmaz, 2020; Liu, Kahathuduwa & Vazsonyi, 2021).

### DNA extraction and genotyping

After a 12-h fast, venous blood samples were collected from all participants using EDTA-containing tubes. Genomic DNA was isolated and purified from the samples using a whole blood genome extraction kit (Beijing Thinkout Sci-Tech Co., Ltd., Beijing, China), and the extracted DNA was stored in a –80 °C freezer. Gene polymorphisms were detected by the SNaPshot method (Larsson et al., 2022). Tag single nucleotide polymorphisms were derived from a Chinese Han population in the Haplotype Map database (National Center for Biotechnology Information, Bethesda, MD, USA) (Sayers et al., 2023). We explored polymorphisms of several major genes that regulate the HPA axis: the *FKBP5* gene (rs1360780, rs3800373, rs9470080, rs4713916, rs3777747, and rs9296158), *CRHR1* gene (rs110402), corticotrophin-releasing hormone type 2 receptor gene (*CRHR2*; rs2267715), and glucocorticoid receptor gene (*NR3C1*; rs41423247). We used SNPs of the above genes, rs1360780 C >T, rs3800373A > C, rs9470080 C > T, rs4713916 G > A, rs3777747 G > A, rs9296158 G > A, rs110402 G > A, rs2267715 G > A and rs41423247 G > C allele combinations, for further haplotype analysis in an attempt to assess the role of haplotypes within the *FKBP5*, *CRHR1*, *CRHR2*, and *NR3C1* genes in susceptibility to sleep disturbances. Table 1 shows the sequences of the primers. The complete sequence is provided in the Supplemental Primer Sequences file.

**Table 1:** Description of primer sequences.

Gene/SNPs	Major/minor alleles	Function	Primer (5′→3′)
*FKBP5*			
rs1360780	C/T	Intron variant, risk-factor	Forward: 5′-GGCATGGGCACTCTGAAAAGAT-3′
			Reverse: 5′-TCTCTTGTGCCAGCAGTAGCAAGT-3′
rs3800373	A/C	3 Prime UTR variant, benign	Forward: 5′-GGCATGGGAAGCTGTCTTCAAC-3′
			Reverse: 5′-CCAGCATTGCTACTGCTCAGCTTC-3′
rs9470080	C/T	Intron variant	Forward: 5′-TCTTTTCCAGGCTATGAATTGACAAA-3′
			Reverse: 5′-TGTGTCCAGCCATGTGCTTTTT-3′
rs4713916	G/A	Intron variant	Forward: 5′-TGGCAACCCTAACCTCTCTGGA-3′
			Reverse: 5′-TGTAGGTTCGGGGTACATGTGAAG-3′
rs3777747	A/G	Intron variant	Forward: 5′-CCGCCTAAGCCTGTTGAGAAGA-3′
			Reverse: 5′-TCCAGTTGTTGGCGTACCTCCT-3′
rs9296158	G/A	Intron variant	Forward: 5′-CACTCGTTCTGTTATACTCATTCCATGC-3′
			Reverse: 5′-AGGCCTGGGCTAGGGGTAATTC-3′
*CRHR1*			
rs110402	G/A	Intron variant	Forward: 5′-AGATCAGCGGATGGTGAAGAGG-3′
			Reverse: 5′-CTTGGCTGCCTAGAACCCTGAC-3′
*CRHR2*			
rs2267715	A/G	Intron variant	Forward: 5′-TCTCTCCCAGCAGGGAAGTTGT-3′
			Reverse: 5′-CTGGAGGGAGTGGGGGTAAACT-3′
*NR3C1*			
rs41423247	G/C	Intron variant	Forward: 5′-GGGGATGAGGTTACGGGGTAGA-3′
			Reverse: 5′-TGCTCACAGGGTTCTTGCCATA-3′

### Confounding factors

It has been demonstrated that some demographic, socioeconomic and lifestyle factors are related to sleep disturbances; thus, they may influence the results of any interaction between sleep disturbances and job stress or HPA axis gene polymorphisms (Wakasugi et al., 2014). In brief, we included age, sex, ethnicity and marital status as confounding factors. In addition, smoking and drinking alcohol were considered potential confounding lifestyle factors.

### Statistical analysis

Statistical analyses were carried out using SPSS version 26.0 (SPSS Inc., Chicago, IL, USA). The ERI and PSQI scores are presented as the mean ± standard deviation (SD). Differences in demographic data between two groups were compared using the chi-squared test for categorical variables. The Hardy‒Weinberg equilibrium (HWE) for the HPA axis gene polymorphisms was tested using a chi-squared goodness-of-fit test. Pearson correlation analysis was used to assess the correlations of job stress with sleep disturbances and job stress dimension scores. After adjusting for sex, age, ethnicity, marital status, smoking status and drinking status as covariates, odds ratios (ORs) and 95% confidence intervals (Levante et al., 2023) were determined for the associations of genotypes and job stress with the risk of sleep disturbances by logistic regression. Bonferroni correction was applied to account for multiple comparisons.

The generalized multifactor dimensionality reduction (GMDR) method is a versatile software for detecting gene–gene and gene–environment interactions underlying complex traits (Xu et al., 2016). In this study, 0.9 GMDR was used to identify the best HPA axis gene × job stress combination, and we used 10-fold cross-validation and 1,000-fold permutation testing. The GMDR provides numerous output parameters, including cross-validation (CV) consistency, testing balanced accuracy, and empirical *P*-values, to assess each selected interaction. The CV consistency score is a measure of the degree of consistency with which the selected interaction is identified as the best model among all possibilities considered (Galimova et al., 2017). We also conducted locus and haplotype analysis for haplotypes associated with sleep disturbances using SHEsis (http://analysis.bio-x.cn/). SHEsis is a powerful software platform for analyses of linkage disequilibrium, haplotype construction, and genetic association at polymorphism loci (Shi & He, 2005). Haplotype analysis was performed to indicate the degree of association between alleles of different SNPs, thus assessing the role of common genotypes in susceptibility to sleep disturbances. All reported *P* values are two-tailed, and those less than 0.05 were considered to indicate statistical significance. G Power software showed that the statistical power of this study was 0.73.

## Results

### Demographic characteristics of the subjects

The general demographic characteristics of the patients in the sleep disturbance group and nonsleep disturbance group are summarized in Table 2. A total of 671 subjects were included in this study, including 269 with sleep disturbances and 402 without sleep disturbances. The incidence of sleep disturbances was 40.09%. We found no significant differences between the two groups in terms of sex, age, ethnicity, marital status, smoking status or drinking status (*P* > 0.05). In addition, of the 671 participants, 121 workers (33.1%) were not experiencing job stress but had sleep disturbances, and 148 (48.5%) were experiencing both job stress and sleep disturbances. There was a significant difference in the distribution of job stress between the two groups (*P* < 0.01).

**Table 2:** Demographic characteristics of 671 participants in non-sleep disturbance and sleep disturbance group.

Variables	N	Non-sleep disturbance (%)	Sleep disturbance (%)	χ^2^	*P*-value
Gender					
Male	363	221 (60.9)	142 (39.1)	0.31	0.58
Female	308	181 (58.8)	127 (41.2)		
Age (years)					
≤30	159	95 (59.7)	64 (40.3)	3.97	0.27
31–40	234	147 (62.8)	87 (37.2)		
41–50	200	109 (54.5)	91 (45.5)		
>51	78	51 (65.4)	28 (34.6)		
Ethnicity					
Han	526	318 (60.5)	208 (39.5)	0.30	0.58
Minority	145	84 (57.9)	61 (42.1)		
Marital status					
Unmarried	118	68 (57.6)	50 (42.4)	2.04	0.36
Married	517	316 (61.1)	201 (38.9)		
Divorced	36	18 (50.0)	18 (50.0)		
Smoking status					
Non-smoker	409	246 (60.1)	163 (39.9)	0.02	0.88
Smoker	262	156 (59.5)	106 (40.5)		
Alcohol status					
Non-drinker	310	183 (59.0)	127 (41.0)	0.19	0.67
Drinker	361	219 (60.7)	142 (39.3)		
Job stress					
Non-job stress	366	245 (66.9)	121 (33.1)	16.57	<0.01
Job stress	305	157 (51.5)	148 (48.5)		

### Correlation between job stress and sleep disturbances

Table 3 shows the correlations among the ERI scores, PSQI scores, and all dimensions of sleep disturbances. When sex, age, ethnicity, marital status, smoking status and drinking status were controlled as covariates, the ERI score was positively correlated with various dimensions of sleep disturbances, including subjective sleep quality and sleep latency (*P* < 0.01). Specifically, overcommitment showed a meaningful positive correlation with sleep medication and the PSQI, with r values of 0.12 and 0.08, respectively. Job effort showed a meaningful positive correlation with sleep medication (r = 0.11). Importantly, there was a positive correlation between the ERI score and PSQI score (r = 0.16, *P* < 0.01), indicating that job stress is related to sleep disturbances, and the greater the job stress is, the greater the risk of sleep disturbances.

**Table 3:** Correlations between the job stress and sleep disturbances and its component scores (*n* = 671).

Variables	Statistical values	Subjective sleep quality	Sleep latency	Sleep duration	Sleep efficiency	Sleep disturbance	Sleep medication	Daytime dysfunction	PSQI
Over-commitment	*r*	0.01	−0.03	−0.01	−0.01	−0.02	0.12	0.00	0.08
	*P*	0.86	0.45	0.88	0.76	0.62	**<0.01**	0.96	**0.04**
Job effort	*r*	0.02	−0.03	−0.01	−0.05	0.05	0.11	0.04	−0.01
	*P*	0.61	0.45	0.74	0.21	0.22	**0.01**	0.26	0.84
Job reward	*r*	0.05	−0.02	0.00	0.01	−0.01	0.05	−0.02	0.01
	*P*	0.19	0.53	0.98	0.90	0.80	0.24	0.59	0.71
ERI	*r*	0.10	0.56	−0.15	0.03	0.03	−0.03	−0.05	0.16
	*P*	**0.01**	**<0.01**	**<0.01**	0.39	0.52	0.49	0.23	**<0.01**

**Note:**

Adjusted for gender, age, ethnicity, marital status, smoking status and alcohol status; r: correlation coefficient, r < 0 indicates negative correlation, and r > 0 indicates positive correlation. Statistically significant *P* value was denoted in bold. There were significant positive correlations between ERI and PSQI (r = 0.16, *P* < 0.01).

### Associations of nine SNPs in HPA axis-related genes with sleep disturbances

The associations of nine SNPs in HPA axis-related genes with sleep disturbances are presented in Table 4. We found that the *FKBP5* rs1360780-TT genotype was associated with increased sleep disturbance risk, with an adjusted OR (95% CI) of 5.34 [3.02–9.44] (*P* = 0.001, Bonferroni-corrected *P* < 0.01). However, the *FKBP5* rs9470080-TT genotype was a protective factor against sleep disturbances, with an adjusted OR (95% CI) of 0.51 [0.28–0.92] (*P* = 0.001, Bonferroni-corrected *P* < 0.01). The *FKBP5* rs1360780-T and rs4713916-A alleles and the *CRHR1* rs110402-G allele were risk factors for sleep disturbances, with adjusted ORs (95% CIs) of 1.75 [1.38–2.22], 1.68 [1.30–2.18] and 1.43 [1.09–1.87], respectively (all *P* = 0.001, Bonferroni-corrected *P* < 0.01). However, the *FKBP5* rs9470080-T allele was a protective factor against sleep disturbances, with an OR (95% CI) of 0.65 [0.51–0.83] (*P* = 0.001, Bonferroni-corrected *P* < 0.01). Haplotype analysis revealed significant differences in the haplotypes between the sleep disturbance group and the nonsleep disturbance group. The C-A-G-A-G-C haplotype was associated with an increased risk of sleep disturbances, and details are provided in the Supplemental File (Table S1).

**Table 4:** Associations of nine SNPs in HPA axis related genes with sleep disturbances.

Genes	SNPs	Genotypes & alleles	Frequencies N (%)		OR (95% CI)	HWE	
			Non-sleep disturbance (*n* = 402)	Sleep disturbance (*n* = 269)		Non-sleep disturbance	Sleep disturbance
*FKBP5*	rs1360780	CC	231 (57.5)	123 (45.7)	1.00	0.88	0.09
		CT	152 (37.8)	93 (34.6)	1.15 [0.82–1.61]		
		TT	19 (4.7)	53 (19.7)	5.24 [2.97–9.24]*		
		C allele	614 (76.4)	339 (63.0)	1.00		
		T allele	190 (23.6)	199 (37.0)	1.75 [1.38–2.22]*		
	rs3800373	AA	234 (58.2)	156 (58.0)	1.00	0.90	0.71
		AC	149 (37.1)	91 (33.8)	0.92 [0.66–1.28]		
		CC	19 (4.7)	22 (8.2)	1.74 [0.91–3.32]		
		A allele	617 (76.7)	403 (74.9)	1.00		
		C allele	187 (23.2)	135 (25.1)	1.11 [0.86–1.43]		
	rs9470080	CC	187 (46.5)	140 (52.0)	1.00	0.92	0.90
		CT	170 (42.3)	112 (41.6)	0.88 [0.64–1.22]		
		TT	45 (11.2)	17 (6.3)	0.51 [0.28–0.92]*		
		C allele	544 (67.7)	392 (72.9)	1.00		
		T allele	260 (32.3)	146 (27.1)	0.65 [0.51–0.83]*		
	rs4713916	GG	256 (63.7)	152 (56.5)	1.00	0.99	0.99
		GA	130 (32.3)	99 (36.8)	1.28 [0.92–1.79]		
		AA	16 (4.0)	18 (6.7)	1.90 (0.94–3.83)		
		G allele	642 (79.9)	403 (74.9)	1.00		
		A allele	162 (20.1)	135 (25.1)	1.68 [1.30–2.18]*		
	rs3777747	AA	66 (16.4)	50 (18.6)	1.00	0.77	0.81
		GA	179 (44.5)	120 (44.6)	0.89 [0.58–1.37]		
		GG	157 (39.1)	99 (36.8)	0.83 [0.53–1.30]		
		A allele	515 (64.1)	220 (40.9)	1.00		
		G allele	289 (35.9)	318 (59.1)	1.13 [0.90–1.41]		
	rs9296158	GG	202 (50.2)	131 (48.7)	1.00	1.00	0.87
		GA	167 (41.5)	109 (40.5)	1.01 [0.72–1.40]		
		AA	33 (8.2)	29 (10.8)	1.36 [0.79–2.34]		
		G allele	571 (71.0)	371 (69.0)	1.00		
		A allele	233 (29.0)	167 (31)	1.13 [0.89–1.43]		
*CRHR1*	rs110402	AA	316 (78.6)	201 (74.7)	1.00	0.73	0.43
		GA	78 (19.4)	57 (21.2)	1.15 [0.78–1.69]		
		GG	8 (2.0)	11 (4.1)	2.16 [0.86–5.47]		
		A allele	94 (11.7)	79 (14.7)	1.00		
		G allele	710 (88.3)	459 (85.3)	1.43 [1.09–1.87]*		
CRHR2	rs2267715	AA	79 (59.5)	61 (22.7)	1.00	0.69	0.89
		GA	183 (34.3)	127 (47.2)	0.90 [0.60–1.35]		
		GG	140 (6.2)	81 (30.1)	0.75 [0.49–1.15]		
		A allele	341 (42.4)	249 (46.3)	1.00		
		G allele	463 (57.6)	289 (53.7)	0.93 [0.74–1.15]		
*NR3C1*	rs41423247	GG	258 (64.2)	168 (62.5)	1.00	0.65	0.81
		GC	122 (30.3)	85 (31.6)	1.07 [0.76–1.50]		
		CC	22 (5.5)	16 (5.9)	1.12 [0.57–2.19]		
		G allele	638 (79.4)	421 (78.3)	1.00		
		C allele	166 (20.6)	117 (21.7)	1.10 [0.84–1.43]		

**Notes:**

Adjusted for gender, age, ethnicity, marital status, smoking status, and alcohol status; the chi-square goodness-of-fit test showed that the genotypic frequencies of nine SNPs in HPA axis related genes in the Non-sleep disturbance group and the sleep disturbance group were consistent with Hardy-Weinberg equilibrium (*P* > 0.05).

* *P* < 0.01.

### Effect of the gene–environment interaction on sleep disturbances

**Figure 1:**
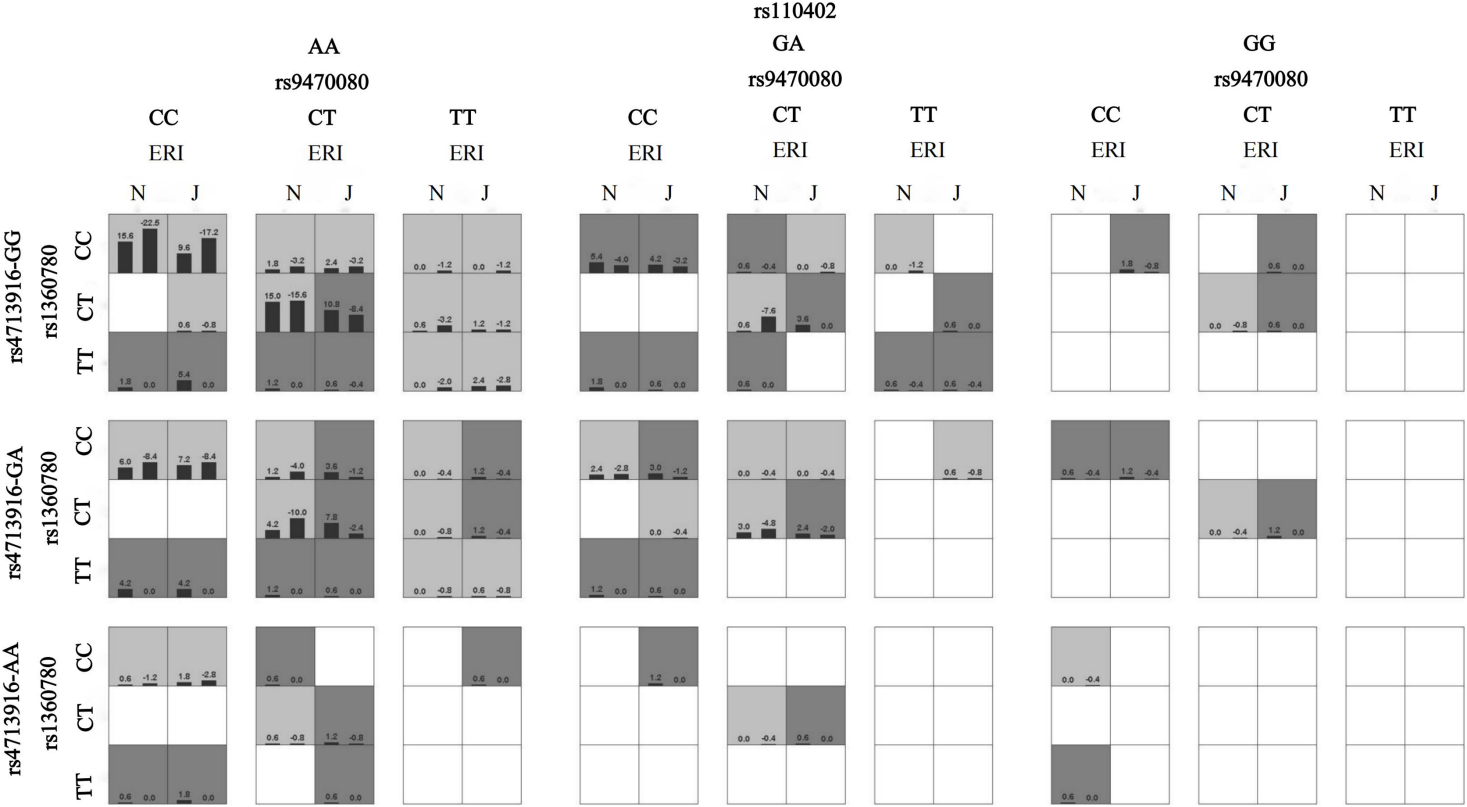
The interaction model between ERI and HPA genes on sleep disturbances. A box represents an interaction combination, the darker the color of the box, the higher the risk of the combination. Bars represent the maximum likelihood estimation of case weights. In the same box, the left column is the positive score of the combination, and the right is the negative score; the higher the positive score, the higher the risk of the combination. In the present study, the dark gray box represents the high sleep disturbances risk factors, and the light gray represents the low sleep disturbances risk factors. N and J denote normal and job stress (ERI > 1), respectively. The best gene-environment interaction model is shown in (the third and fourth columns of the second row). Under the job stress (J) (the fourth column of the second row), the rs1368780-CT, rs4713916-GG, and rs9470080-CT and the rs110402-AA interacted with the highest scores, and the positive scores were greater than the negative scores (10.8, −8.4), which indicates that under job stress, the subjects with the *FKBP5* rs1368780-CT, rs4713916-GG, and rs9470080-CT genotypes and the *CRHR1* rs110402-AA genotype had the highest sleep disturbance risk.

When sex, age, ethnicity, marital status, smoking status and drinking status were controlled as covariates, the best gene‒environment interaction models were determined by GMDR analysis (Table 5). These models showed a significant effect of the interaction between HPA axis genes and job stress on sleep disturbances. The model had the maximum cross-validation consistency coefficient (10/10), and the accuracies of the training set and testing set were 0.68 and 0.60, respectively. ERI × rs1360780 × rs947008 × rs4713916 × rs110402 was considered the best interaction model because it contained the most SNPs among the models that met the best model criteria. This suggests that the best interaction model was the interaction between job stress and *FKBP5* rs1360780, rs9470080, and rs4713916 genotypes and the *CRHR1* rs110402 genotype. Furthermore, we also found that under job stress, the subjects with *FKBP5* rs1368780-CT, rs4713916-GG, and rs9470080-CT genotypes and the *CRHR1* rs110402-AA genotype had the greatest risk of sleep disturbances (Fig. 1). In addition, we analyzed the HPA axis gene‒gene interactions, and the results showed that rs1360780 × rs947008 × rs110402 was the best gene‒gene interaction model among the nine SNPs in the genes related to the HPA axis (Table S2 and Fig. S1).

**Table 5:** Best gene-environment interaction models, as identified by GMDR.

Model	Training accuracy (%)	Testing accuracy (%)	Cross-validation consistency	*P*-value
ERI	0.58	0.56	8/10	0.17
ERI × rs1360780	0.62	0.61	10/10	**0.01**
ERI × rs1360780 × rs947008	0.64	0.60	4/10	**<0.01**
ERI × rs1360780 × rs947008 × rs110402	0.66	0.64	10/10	**<0.01**
ERI × rs1360780 × rs947008 × rs4713916 × rs110402	0.68	0.60	10/10	**<0.01**

**Note:**

Adjusted for gender, age, ethnicity, marital status, smoking status and alcohol status. The best interaction model was selected based on the balance test error of the 1/10 test sample, the accuracy of the cross-validation and *P*-value and more SNPs included in the model, suggest that ERI × rs1360780 × rs947008 × rs4713916 × rs110402 is the best interaction model (Cross-Validation Consistency:10/10, *P* < 0.01). Statistically significant *P* value was denoted in bold.

## Discussion

To our knowledge, this is the first study to investigate the associations among multiple HPA axis gene polymorphisms, job stress, and their interactions with sleep disturbances. Our study has three main findings. (a) After controlling for confounding factors such as sex, age and ethnicity, job stress was correlated with sleep disturbances. (b) *FKBP5* rs1360780-T and rs4713916-A alleles and the *CRHR1* rs110402-G allele were associated with the risk of sleep disturbances. However, the *FKBP5* rs9470080-T allele was a protective factor against sleep disturbances. (c) GMDR analysis showed that in individuals under job stress, the risk of sleep disturbances was the highest for the *FKBP5* rs1368780-CT, rs4713916-GG, and rs9470080-CT genotypes and the *CRHR1* rs110402-AA genotype.

In this study, we found that the greater the level of job stress experienced, the worse the sleep quality. Consistent with previous studies, many studies have shown that high job stress is associated with a greater risk of insomnia (Deguchi et al., 2017; Yang et al., 2018; Wang et al., 2022a). In addition, overcommitment was also meaningfully and positively correlated with the PSQI score. This result also suggested that overcommitment and job stress may be related to sleep disturbances (Yoshioka et al., 2013; Wang et al., 2020). Lallukka et al. reached the same conclusion (Lallukka et al., 2014). Job stress is a very influential environmental factor for sleep (Gosling et al., 2014). There is evidence that the basal levels of cortisol are elevated in individuals experiencing job stress, and the HPA axis of people experiencing job stress may release the cortisol that causes sleep disturbances (Fogelman & Canli, 2018; Rotvig et al., 2019). In addition, Birch & Vanderheyden (2022) explored how job stress mediates stress-induced insomnia by regulating the glucocorticoid signaling pathway in brain astrocytes. This evidence suggests that job stress interferes with normal sleep and even increases the risk of sleep disturbances by activating the HPA axis.

Consistent with previous results, our study also revealed correlations between several major HPA axis regulatory genes and sleep disturbances. This result indicated that individuals with *FKBP5* rs1360780-T and rs4713916-A alleles and the *CRHR1* rs110402-G allele had a greater risk of sleep disturbance. This finding is in line with a study by White et al. (2012) that showed that the interaction between *FKBP5* minor alleles (including rs1360780-T and rs4713916-A alleles) and emotional neglect may increase the risk of stress-related disorders such as sleep disturbances. In addition, previous studies have shown that participants with the *CRHR1* rs110402-A allele had higher cortisol levels 15 min poststress, implying a risk of sleep disturbances in the future (Weeger et al., 2020; Nold et al., 2021). A meta-analysis showed that individuals exposed to stress and carrying the rs1360780-T allele and rs3800373-C allele had significantly shorter sleep durations and greater risks of stress-related diseases (Wang, Shelton & Dwivedi, 2018). Moreover, a study by Maguire et al. (2020) suggested that stress-related alterations in HPA axis genes in individuals with PTSD may contribute to sleep difficulties. We also found a protective effect of the *FKBP5* rs9470080-TT genotype against sleep disturbances, which is different from the results of another study (Li et al., 2019). We believe that these inconsistent results may be caused by different types of stress or stressors. Previous findings have been based primarily on posttraumatic stress in earthquake survivors. Another possible explanation for the differences is the questionnaires and evaluation criteria used.

Our findings provide new insights into the effects of gene‒environment interactions on sleep disturbances. We found that the HPA axis gene × job stress interaction strongly affects sleep disturbances. More importantly, the GMDR results showed that individuals with the *FKBP5* rs1360780-CC genotype, rs9470080-CC genotype and *CRHR1* rs110402-AA genotype have the highest risk of sleep disturbances under job stress. Previous studies have also revealed effects of gene‒environment interactions on sleep disturbances. For example, Zimmermann et al. (2011) reported that individuals carrying risk alleles of two *FKBP5* SNPs (rs3000377 and rs47139611) have the highest risk of reduced sleep quality if they have experienced adverse life events. Similar results were found for the interaction between childhood trauma and risk alleles of these SNPs (Bevilacqua et al., 2012). Similarly, He et al. (2019) investigated 712 participants in a large general hospital in Beijing, and the results suggested that when experiencing work-related stress, individuals with the *CRHR1* rs110402-A allele may experience reduced sleep quality. In summary, our study provides evidence that the HPA axis gene × job stress interaction may play an important role in sleep disturbances. Furthermore, according to previous research, the gene × stress interaction can be explained by the diathesis-stress model (Belsky & Pluess, 2009). The model suggests that individuals with “risk-associated genes” are prone to stress-related diseases such as sleep disturbances when confronted with stress or adverse environments, while individuals with “resilient-associated genes” are not affected (Monroe & Simons, 1991; Shao et al., 2018). As diathesis-stress research has highlighted, the interaction of *FKBP5* variants with trauma and adverse environments has been found to confer risk for several psychopathological phenotypes (Zannas et al., 2016). In this study, the *FKBP5* rs1360780-CC and rs9470080-CC genotypes and the *CRHR1* rs110402-AA genotype may be risk factors for susceptibility to stressful environments, supporting the diathesis-stress model. Therefore, to reduce the risk of sleep disturbances, individuals with genetic susceptibility should avoid or reduce job stress as much as possible.

This study has several strengths. This is the first study to examine the effects of multiple gene polymorphisms and job stress on sleep disturbances from the perspective of the HPA axis and to determine a haplotype that increases the power to detect genetic associations (Aziz et al., 2021). Haplotype analysis can assess the role of different genotypes of the target gene in susceptibility to sleep disturbances. Furthermore, GMDR was used to investigate the pattern of gene × environment interactions, as it recognizes interactions between multiple loci or environmental factors (Hou et al., 2019). However, this research still has some limitations that can be addressed in future studies. First, the evaluation of sleep disturbances was entirely based on the PSQI, and the evaluation of job stress was based exclusively on the ERI, which are subjective questionnaires that are prone to produce false positive results, which may have affected the accuracy of the results. Second, there are different sources of sample bias, including reaction bias (*e.g*., subjects with poor sleep quality may be more inclined to complete the study than those with good sleep quality) and sample-selection bias (*e.g*., first-line railway workers are apt to work long hours in stressful environments). Finally, a cross-sectional design was used; thus, we could not examine the causality of the HPA axis gene × job stress interaction in the development of sleep disturbances. In future research, longitudinal designs should be used to further study this causal relationship, as well as experimental methods to measure subjects’ sleep disturbances and job stress to provide experimental support for the results of the present study. Importantly, we will incorporate transcriptomic, epigenomic, or HPA axis activity data to conduct more in-depth studies. The results of this study suggest that individuals who carry risk alleles may be at increased risk for sleep disturbances if they are under job stress. This study provides a reliable basis for formulating strategies to reduce employees’ job stress and improve sleep quality. This study suggested that industries should pay attention to the occupationally stressful situations of their workers by reducing the incidence of occupational stress (reduction of working hours and tasks, work incentives and support) and thus reducing the incidence of sleep disturbances.

## Conclusions

This is the first study to investigate the effect of the interaction between job stress and HPA axis gene polymorphisms on sleep disturbances in railway frontline workers. The present study revealed that 48.5% of workers experienced both job stress and sleep disturbances. As the main effect of sleep quality, job stress was found to increase the risk of sleep disturbances. The *FKBP5* rs1360780-T and rs4713916-A alleles and the *CRHR1* rs110402-G allele were also risk factors for sleep disturbances. More importantly, the GMDR results showed that the interactions of SNPs with job stress increased the risk of sleep disturbances, which is the core conclusion of our study. These findings provide new insight into the correlation between job stress and HPA axis gene polymorphisms and their interaction with sleep disturbances.

## Supplemental Information

10.7717/peerj.17119/supp-1 Supplemental Information 1 The interaction model between 9 HPA genes SNPs on sleep disturbances. A box represents an interaction combination, the darker the color of the box, the higher the risk of the combination. Bars represent the maximum likelihood estimation of case weights. In the same box, the left column is the positive score of the combination, and the right is the negative score; the higher the positive score, the higher the risk of the combination. In the present study, the dark gray box represents the high sleep disturbances risk factors, and the light gray represents the low sleep disturbances risk factors. The best gene-gene interaction model is shown in (Second row, second column), which suggests that the subjects with the *FKBP5* rs9470080-CT and rs1360780-CT genotypes and the *CRHR1* rs110402-AA genotype had the highest sleep disturbance risk.

10.7717/peerj.17119/supp-2 Supplemental Information 2 Haplotype analysis of FKBP5 gene. Loci chosen for hap-analysis: rs1360780, rs4713916, rs3777747, rs3800373, rs9296158, rs9470080. All alleles of the above SNPs were analyzed by haplotype, using the first allele of each SNP as the reference standard (results are shown in Table 4, *e.g*., OR (95% CI) = 1.00 for rs1360780-C allele), and alleles with a frequency of less than 0.03 in controls and cases were excluded. Global chi2 is 123.439682, while df = 10, *P* **<** 0.01. Loci chosen for hap-analysis: rs1360780, rs4713916, rs3777747, rs3800373, rs9296158, rs9470080.

10.7717/peerj.17119/supp-3 Supplemental Information 3 Best gene-gene interaction models, as identified by GMDR. Adjusted for gender, age, ethnicity, marital status, smoking status and alcohol status. The best interaction model was selected based on the balance test error of the 1/10 test sample, the accuracy of the cross-validation and P-value, suggest that rs1360780 ×rs947008 ×rs110402 is the best interaction model (Cross-Validation Consistency:10/10, *P* < 0.001). Statistically significant P value was denoted in bold.

10.7717/peerj.17119/supp-4 Supplemental Information 4 Raw Data. Scores for all questionnaires and for all genes. These data were used to analyze the interaction of genes and job stress on sleep disturbance.

10.7717/peerj.17119/supp-5 Supplemental Information 5 Codebook.

10.7717/peerj.17119/supp-6 Supplemental Information 6 STROBE checklist v4 cross-sectional.

10.7717/peerj.17119/supp-7 Supplemental Information 7 Gene Sequence.

10.7717/peerj.17119/supp-8 Supplemental Information 8 Supplementary Primer Sequences.

## Additional Information and Declarations

### Data Availability

The following information was supplied regarding data availability: The raw measurements are available in the Supplemental File.

## References

[ref-1] Albu S, Romanowski CP, Letizia Curzi M, Jakubcakova V, Flachskamm C, Gassen NC, Hartmann J, Schmidt MV, Schmidt U, Rein T, Holsboer F, Hausch F, Paez‐Pereda M, Kimura M (2014). Deficiency of FK506-binding protein (FKBP) 51 alters sleep architecture and recovery sleep responses to stress in mice. Journal of Sleep Research.

[ref-2] Aziz NA, Taib WRW, Kharolazaman NK, Ismail I, Al-Jamal HAN, Jamil NW-AWA, Esa E, Ibrahim H (2021). Evidence of new intragenic HBB haplotypes model for the prediction of beta-thalassemia in the Malaysian population. Scientific Reports.

[ref-3] Bakker E, Tian K, Mutti L, Demonacos C, Schwartz J-M, Krstic-Demonacos M, Rzhetsky A (2017). Insight into glucocorticoid receptor signalling through interactome model analysis. PLOS Computational Biology.

[ref-4] Basu S, Qayyum H, Mason S (2017). Occupational stress in the ED: a systematic literature review. Emergency Medicine Journal.

[ref-5] Belsky J, Pluess M (2009). Beyond diathesis stress: differential susceptibility to environmental influences. Psychological Bulletin.

[ref-6] Bevilacqua L, Carli V, Sarchiapone M, George DK, Goldman D, Roy A, Enoch MA (2012). Interaction between FKBP5 and childhood trauma and risk of aggressive behavior. Archives of General Psychiatry.

[ref-7] Birch JN, Vanderheyden WM (2022). The molecular relationship between stress and insomnia. Advanced Biology.

[ref-8] Blom V, Kallings LV, Ekblom B (2020). Self-reported general health, overall and work-related stress, loneliness, and sleeping problems in 335,625 Swedish adults from 2000 to 2016. International Journal of Environmental Research and Public Health.

[ref-9] Brummett BH, Krystal AD, Ashley-Koch A, Kuhn CM, Züchner S, Siegler IC, Barefoot JC, Ballard EL, Gwyther LP, Williams RB (2007). Sleep quality varies as a function of 5-HTTLPR genotype and stress. Psychosomatic Medicine.

[ref-10] Buckley TM, Schatzberg AF (2005). On the interactions of the hypothalamic-pituitary-adrenal (HPA) axis and sleep: normal HPA axis activity and circadian rhythm, exemplary sleep disorders. The Journal of Clinical Endocrinology & Metabolism.

[ref-11] Buysse DJ, Reynolds CF, Monk TH, Berman SR, Kupfer DJ (1989). The pittsburgh sleep quality index: a new instrument for psychiatric practice and research. Psychiatry Research.

[ref-12] Chaput JP, Yau J, Rao DP, Morin CM (2018). Prevalence of insomnia for Canadians aged 6 to 79. Health Reports.

[ref-13] Choi B, Ko S, Dobson M, Schnall PL, Garcia-Rivas J, Israel L, Baker D (2014). Short-term test-retest reliability of the job content questionnaire and effort-reward imbalance questionnaire items and scales among professional firefighters. Ergonomics.

[ref-14] de Feijter M, Katimertzoglou A, Tiemensma J, Ikram MA, Luik AI (2022). Polysomnography-estimated sleep and the negative feedback loop of the hypothalamic-pituitary-adrenal (HPA) axis. Psychoneuroendocrinology.

[ref-15] Deguchi Y, Iwasaki S, Ishimoto H, Ogawa K, Fukuda Y, Nitta T, Mitake T, Nogi Y, Inoue K (2017). Relationships between temperaments, occupational stress, and insomnia among Japanese workers. PLOS ONE.

[ref-16] D’Ettorre G, Pellicani V, Caroli A, Greco M (2020). Shift work sleep disorder and job stress in shift nurses: implications for preventive interventions. Medicina Del Lavoro.

[ref-17] Federenko IS, Nagamine M, Hellhammer DH, Wadhwa PD, Wüst S (2004). The heritability of hypothalamus pituitary adrenal axis responses to psychosocial stress is context dependent. The Journal of Clinical Endocrinology and Metabolism.

[ref-18] Fogelman N, Canli T (2018). Early life stress and cortisol: a meta-analysis. Hormones and Behavior.

[ref-19] Foley P, Kirschbaum C (2010). Human hypothalamus-pituitary-adrenal axis responses to acute psychosocial stress in laboratory settings. Neuroscience & Biobehavioral Reviews.

[ref-20] Galimova E, Rätsep R, Traks T, Kingo K, Escott-Price V, Kõks S (2017). Interleukin-10 family cytokines pathway: genetic variants and psoriasis. British Journal of Dermatology.

[ref-21] Gerritsen L, Milaneschi Y, Vinkers CH, van Hemert AM, van Velzen L, Schmaal L, Penninx BWJH (2017). HPA axis genes, and their interaction with childhood maltreatment, are related to cortisol levels and stress-related phenotypes. Neuropsychopharmacology.

[ref-22] Gosling JA, Batterham PJ, Glozier N, Christensen H (2014). The influence of job stress, social support and health status on intermittent and chronic sleep disturbance: an 8-year longitudinal analysis. Sleep Medicine.

[ref-29] Hämmig O (2020). Work- and stress-related musculoskeletal and sleep disorders among health professionals: a cross-sectional study in a hospital setting in Switzerland. BMC Musculoskeletal Disorders.

[ref-23] Halonen JI, Lallukka T, Pentti J, Stenholm S, Rod NH, Virtanen M, Salo P, Kivimäki M, Vahtera J (2017). Change in job strain as a predictor of change in insomnia symptoms: analyzing observational data as a non-randomized pseudo-trial. Sleep.

[ref-24] He SC, Wu S, Du XD, Jia Q, Wang C, Wu F, Ning Y, Wang D, Wang L, Zhang XY (2019). Interactive effects of corticotropin-releasing hormone receptor 1 gene and work stress on burnout in medical professionals in a Chinese Han population. Journal of Affective Disorders.

[ref-25] Herr RM, Barrech A, Riedel N, Gündel H, Angerer P, Li J (2018). Long-term effectiveness of stress management at work: effects of the changes in perceived stress reactivity on mental health and sleep problems seven years later. International Journal of Environmental Research and Public Health.

[ref-26] Hirotsu C, Tufik S, Andersen ML (2015). Interactions between sleep, stress, and metabolism: from physiological to pathological conditions. Sleep Science.

[ref-27] Hou TT, Lin F, Bai S, Cleves MA, Xu H‐M, Lou X‐Y (2019). Generalized multifactor dimensionality reduction approaches to identification of genetic interactions underlying ordinal traits. Genetic Epidemiology.

[ref-28] Huang C, Li J, Lu L, Ren X, Li Y, Huang Q, Lan Y, Wang Y (2014). Interaction between serotonin transporter gene-linked polymorphic region (5-HTTLPR) and job-related stress in insomnia: a cross-sectional study in Sichuan. Sleep Medicine.

[ref-30] Jiang Y, Liu B, Wu C, Gao X, Lu Y, Lian Y, Liu J (2020). Dopamine receptor D2 gene (DRD2) polymorphisms, job stress, and their interaction on sleep dysfunction. International Journal of Environmental Research and Public Health.

[ref-31] Juster RP, McEwen BS (2015). Sleep and chronic stress: new directions for allostatic load research. Sleep Medicine.

[ref-32] Kadier K, Dilixiati D, Ainiwaer A (2023). Analysis of the relationship between sleep-related disorder and systemic immune-inflammation index in the US population. BMC Psychiatry.

[ref-33] Khamisa N, Peltzer K, Ilic D, Oldenburg B (2016). Work related stress, burnout, job satisfaction and general health of nurses: a follow-up study. International Journal of Nursing Practice.

[ref-34] Kucharczyk ER, Morgan K, Hall AP (2012). The occupational impact of sleep quality and insomnia symptoms. Sleep Medicine Reviews.

[ref-35] Kudielka BM, Wüst S (2010). Human models in acute and chronic stress: assessing determinants of individual hypothalamus-pituitary-adrenal axis activity and reactivity. Stress-the International Journal on the Biology of Stress.

[ref-36] Lallukka T, Ferrie JE, Kivimäki M, Shipley MJ, Sekine M, Tatsuse T, Pietiläinen O, Rahkonen O, Marmot MG, Lahelma E (2014). Conflicts between work and family life and subsequent sleep problems among employees from Finland, Britain, and Japan. International Journal of Behavioral Medicine.

[ref-37] Larsson L, Bergenstråhle L, He M, Andrusivova Z, Lundeberg J (2022). SnapShot: spatial transcriptomics. Cell.

[ref-38] Levante A, Petrocchi S, Bianco F, Castelli I, Lecciso F (2023). Teachers during the COVID-19 Era: the mediation role played by mentalizing ability on the relationship between depressive symptoms, anxious trait, and job burnout. International Journal of Environmental Research and Public Health.

[ref-39] Li G, Wang L, Zhang K, Cao C, Fang R, Liu P, Luo S, Zhang X (2019). FKBP5 genotype linked to combined PTSD-depression symptom in chinese earthquake survivors. The Canadian Journal of Psychiatry.

[ref-40] Lian Y, Xiao J, Wang Q, Ning L, Guan S, Ge H, Li F, Liu J (2014). The relationship between glucocorticoid receptor polymorphisms, stressful life events, social support, and post-traumatic stress disorder. BMC Psychiatry.

[ref-41] Linh TTD, Ho DKN, Nguyen NN, Hu C-J, Yang C-H, Wu D (2023). Global prevalence of post-COVID-19 sleep disturbances in adults at different follow-up time points: a systematic review and meta-analysis. Sleep Medicine Reviews.

[ref-42] Linton SJ, Kecklund G, Franklin KA, Leissner LC, Sivertsen B, Lindberg E, Svensson AC, Hansson SO, Sundin Ö, Hetta J, Björkelund C, Hall C (2015). The effect of the work environment on future sleep disturbances: a systematic review. Sleep Medicine Reviews.

[ref-43] Liu D, Kahathuduwa C, Vazsonyi AT (2021). The pittsburgh sleep quality index (PSQI): psychometric and clinical risk score applications among college students. Psychological Assessment.

[ref-44] Maguire DG, Ruddock MW, Milanak ME, Moore T, Cobice D, Armour C (2020). Sleep, a governor of morbidity in PTSD: a systematic review of biological markers in PTSD-related sleep disturbances. Nature and Science of Sleep.

[ref-45] Mahon PB, Zandi PP, Potash JB, Nestadt G, Wand GS (2013). Genetic association of FKBP5 and CRHR1 with cortisol response to acute psychosocial stress in healthy adults. Psychopharmacology (Berl).

[ref-46] Matosin N, Halldorsdottir T, Binder EB (2018). Understanding the molecular mechanisms underpinning gene by environment interactions in psychiatric disorders: the FKBP5 model. Biological Psychiatry.

[ref-47] Miyachi T, Nomura K, Minamizono S, Sakai K, Iwata T, Sugano Y, Sawaguchi S, Takahashi K, Mishima K (2021). Factors associated with insomnia among truck drivers in Japan. Nature and Science of Sleep.

[ref-48] Mollayeva T, Thurairajah P, Burton K, Mollayeva S, Shapiro CM, Colantonio A (2016). The Pittsburgh sleep quality index as a screening tool for sleep dysfunction in clinical and non-clinical samples: a systematic review and meta-analysis. Sleep Medicine Reviews.

[ref-49] Monroe SM, Simons AD (1991). Diathesis-stress theories in the context of life stress research: implications for the depressive disorders. Psychological Bulletin.

[ref-50] Morin CM, Jarrin DC (2022). Epidemiology of insomnia: prevalence, course, risk factors, and public health burden. Sleep Medicine Clinics.

[ref-51] Nicolaides NC, Vgontzas AN, Kritikou I, Feingold KR, Anawalt B, Boyce A (2000). HPA axis and sleep. Endotext.

[ref-52] Nold V, Portenhauser M, Del Prete D, Blasius A, Harris I, Koros E, Peleh T, Hengerer B, Kolassa I-T, Slezak M, Allers KA (2022). Impact of Fkbp5 × early life adversity × sex in humanised mice on multidimensional stress responses and circadian rhythmicity. Molecular Psychiatry.

[ref-53] Nold V, Richter N, Hengerer B, Kolassa I‐T, Allers KA (2021). FKBP5 polymorphisms induce differential glucocorticoid responsiveness in primary CNS cells-first insights from novel humanized mice. European Journal of Neuroscience.

[ref-54] Nordentoft M, Rod NH, Bonde JP, Bjorner JB, Cleal B, Madsen IEH, Magnusson Hanson LL, Nexo MA, Sterud T, Rugulies R (2020). Changes in effort-reward imbalance at work and risk of onset of sleep disturbances in a population-based cohort of workers in Denmark. Sleep Medicine X.

[ref-55] Normann C, Buttenschøn HN (2020). Gene-environment interactions between HPA-axis genes and childhood maltreatment in depression: a systematic review. Acta Neuropsychiatrica.

[ref-56] Ota A, Masue T, Yasuda N, Tsutsumi A, Mino Y, Ohara H, Ono Y (2009). Psychosocial job characteristics and insomnia: a prospective cohort study using the demand-control-support (DCS) and effort-reward imbalance (ERI) job stress models. Sleep Medicine.

[ref-57] Rajaratnam SMW, Barger LK, Lockley SW, Shea SA, Wang W, Landrigan CP, O’Brien CS, Qadri S, Sullivan JP, Cade BE, Epstein LJ, White DP, Czeisler CA, Harvard Work Hours, Health and Safety Group (2011). Sleep disorders, health, and safety in police officers. JAMA.

[ref-58] Ramar K, Malhotra RK, Carden KA, Martin JL, Abbasi-Feinberg F, Aurora RN, Kapur VK, Olson EJ, Rosen CL, Rowley JA, Shelgikar AV, Trotti LM (2021). Sleep is essential to health: an American academy of sleep medicine position statement. Journal of Clinical Sleep Medicine.

[ref-59] Rotvig DH, Bauer JØ, Eller NH, Jørgensen MB (2019). [Work-related stress and the hypothalamic-pituitary-adrenal axis]. Ugeskr Laeger.

[ref-60] Sayers EW, Bolton EE, Brister JR, Canese K, Chan J, Comeau D C, Farrell C M, Feldgarden M, Fine AM, Funk K, Hatcher E, Kannan S, Kelly C, Kim S, Klimke W, Landrum M J, Lathrop S, Lu Z, Madden T L, Malheiro A, Marchler-Bauer A, Murphy T D, Phan L, Pujar S, Rangwala S H, Schneider V A, Tse T, Wang J, Ye J, Trawick B W, Pruitt K D, Sherry S T (2023). Database resources of the national center for biotechnology information in 2023. Nucleic Acids Research.

[ref-61] Shao D, Zhang HH, Long ZT, Li J, Bai HY, Li JJ, Cao FL (2018). Effect of the interaction between oxytocin receptor gene polymorphism (rs53576) and stressful life events on aggression in Chinese Han adolescents. Psychoneuroendocrinology.

[ref-62] Shi YY, He L (2005). SHEsis, a powerful software platform for analyses of linkage disequilibrium, haplotype construction, and genetic association at polymorphism loci. Cell Research.

[ref-63] Shi L, Lu ZA, Que JY, Huang X-L, Liu L, Ran M-S, Gong Y-M, Yuan K, Yan W, Sun Y-K, Shi J, Bao Y-P, Lu L (2020). Prevalence of and risk factors associated with mental health symptoms among the general population in China during the Coronavirus Disease 2019 Pandemic. JAMA Network Open.

[ref-64] Siegrist J, Li J (2017). Work Stress and altered biomarkers: a synthesis of findings based on the effort-reward imbalance model. International Journal of Environmental Research and Public Health.

[ref-65] Uehli K, Mehta AJ, Miedinger D, Hug K, Schindler C, Holsboer-Trachsler E, Leuppi JD, Künzli N (2014). Sleep problems and work injuries: a systematic review and meta-analysis. Sleep Medicine Reviews.

[ref-66] Wakasugi M, Kazama JJ, Narita I, Iseki K, Moriyama T, Yamagata K, Fujimoto S, Tsuruya K, Asahi K, Konta T, Kimura K, Kondo M, Kurahashi I, Ohashi Y, Watanabe T (2014). Association between combined lifestyle factors and non-restorative sleep in Japan: a cross-sectional study based on a Japanese health database. PLOS ONE.

[ref-67] Wang C, Liu J, Li Z, Ji L, Wang R, Song H, Mao Y, Bishwajit G, Zhang B, Tang S (2019). Predictor of sleep difficulty among community dwelling older populations in 2 African settings. Medicine (Baltimore).

[ref-68] Wang MF, Shao P, Wu C, Zhang LY, Zhang LF, Liang J, Du J (2022a). The relationship between occupational stressors and insomnia in hospital nurses: the mediating role of psychological capital. Frontiers in Psychology.

[ref-69] Wang Q, Shelton RC, Dwivedi Y (2018). Interaction between early-life stress and FKBP5 gene variants in major depressive disorder and post-traumatic stress disorder: a systematic review and meta-analysis. Journal of Affective Disorders.

[ref-70] Wang A, Wei Z, Yuan H, Zhu Y, Peng Y, Gao Z, Liu Y, Shen J, Xu H, Guan J, Yin S, Liu F, Li X (2023). FKBP5 genetic variants are associated with respiratory- and sleep-related parameters in Chinese patients with obstructive sleep apnea. Frontiers in Neuroscience.

[ref-71] Wang C, Xu XH, Cui XY, Liu XD, Li T, Li S, Liu YM, Liu XM, Zhou H, Li J, Niu DS (2020). [The influencing effects of effort reward imbalance on sleep disorders among metro staff in Guangzhou]. Zhonghua Lao Dong Wei Sheng Zhi Ye Bing Za Zhi.

[ref-72] Wang Y, Zhao M, Li P, Wu C, Lv Y, Jiang Y (2022b). Gene-environment interaction between circadian clock gene polymorphisms and job stress on the risk of sleep disturbances. Psychopharmacology (Berl).

[ref-73] Weeger J, Ising M, Müller-Myhsok B, Uhr M, Schmidt U, Steiger A (2020). Salivary cortisol response to psychosocial stress in the late evening depends on CRHR1 genotype. Psychoneuroendocrinology.

[ref-74] Weitzman ED, Zimmerman JC, Czeisler CA, Ronda J (1983). Cortisol secretion is inhibited during sleep in normal man. The Journal of Clinical Endocrinology & Metabolism.

[ref-75] White MG, Bogdan R, Fisher PM, Muñoz KE, Williamson DE, Hariri AR (2012). FKBP5 and emotional neglect interact to predict individual differences in amygdala reactivity. Genes Brain and Behavior.

[ref-76] Wu T, Jia X, Shi H, Niu J, Yin X, Xie J, Wang X (2021). Prevalence of mental health problems during the COVID-19 pandemic: a systematic review and meta-analysis. Journal of Affective Disorders.

[ref-77] Xu HM, Xu LF, Hou TT, Luo LF, Chen GB, Sun XW, Lou XY (2016). GMDR: versatile software for detecting gene-gene and gene-environ- ment interactions underlying complex traits. Current Genomics.

[ref-78] Yang B, Wang Y, Cui F, Huang T, Sheng P, Shi T, Huang C, Lan Y, Huang Y-N (2018). Association between insomnia and job stress: a meta-analysis. Sleep and Breathing.

[ref-79] Yilmaz M (2020). Evaluation of sleep disorders in nonmetastatic breast cancer patients based on pittsburgh sleep quality index. Journal of Cancer Research and Therapeutics.

[ref-80] Yoshioka E, Saijo Y, Kita T, Satoh H, Kawaharada M, Kishi R (2013). Effect of the interaction between employment level and psychosocial work environment on insomnia in male Japanese public service workers. International Journal of Behavioral Medicine.

[ref-81] Zannas AS, Wiechmann T, Gassen NC, Binder EB (2016). Gene-stress-epigenetic regulation of FKBP5: clinical and translational implications. Neuropsychopharmacology.

[ref-82] Zhang H, Khan A, Rzhetsky A (2022). Gene-environment interactions explain a substantial portion of variability of common neuropsychiatric disorders. Cell Reports Medicine.

[ref-83] Zimmermann P, Brückl T, Nocon A, Pfister H, Binder EB, Uhr M, Lieb R, Moffitt TE, Caspi A, Holsboer F, Ising M (2011). Interaction of FKBP5 gene variants and adverse life events in predicting depression onset: results from a 10-year prospective community study. American Journal of Psychiatry.

[ref-84] Zwicker A, Denovan-Wright EM, Uher R (2018). Gene-environment interplay in the etiology of psychosis. Psychological Medicine.

