# Supplementary figures and images for: Gene‒environment interaction effect of hypothalamic‒pituitary‒adrenal axis gene polymorphisms and job stress on the risk of sleep disturbances
Source: PeerJ. 2024 Mar 20;12:e17119. doi: 10.7717/peerj.17119 (PMC10960531; doi:10.7717/peerj.17119)

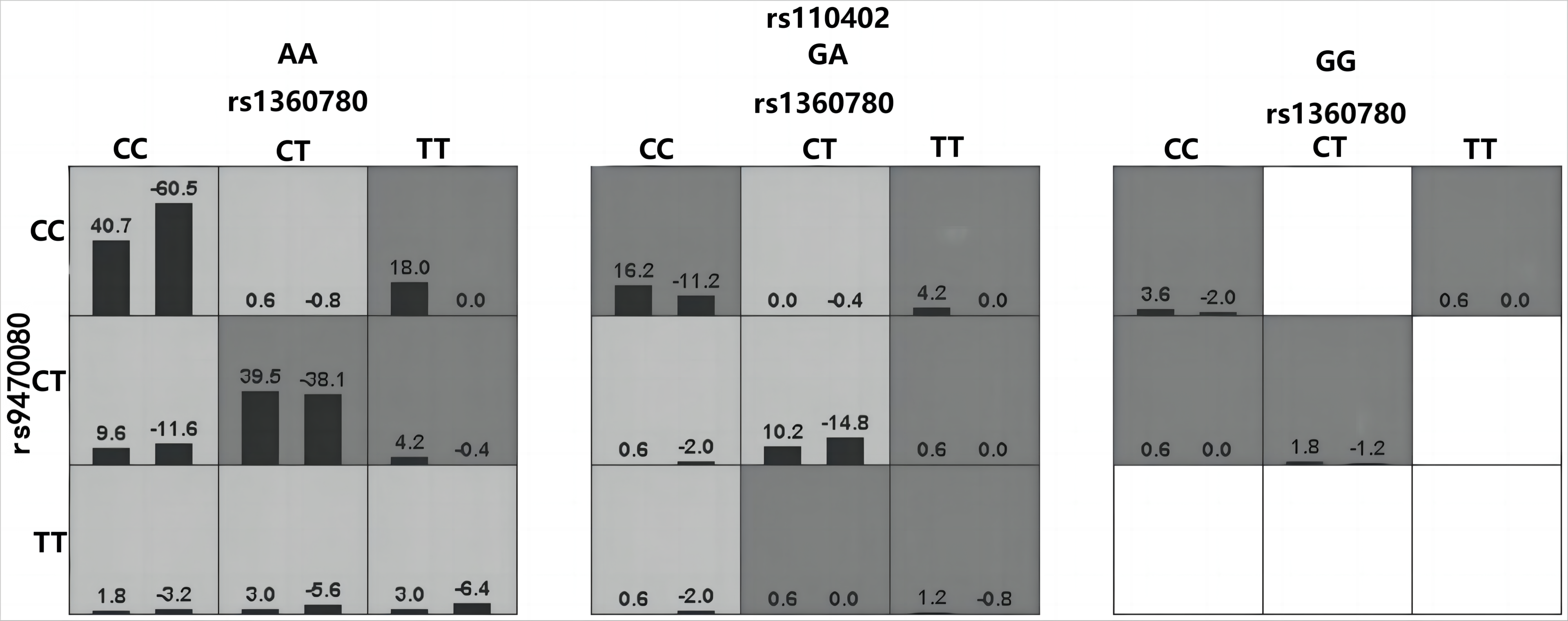

Supplement: Supplemental Information 1 — A box represents an interaction combination, the darker the color of the box, the higher the risk of the combination. Bars represent the maximum likelihood estimation of case weights. In the same box, the left column is the positive score of the combination, and the right is the negative score; the higher the positive score, the higher the risk of the combination. In the present study, the dark gray box represents the high sleep disturbances risk factors, and the light gray represents the low sleep disturbances risk factors. The best gene-gene interaction model is shown in (Second row, second column), which suggests that the subjects with the FKBP5 rs9470080-CT and rs1360780-CT genotypes and the CRHR1 rs110402-AA genotype had the highest sleep disturbance risk. [file peerj-12-17119-s001.png]
