# Supplementary material for: Gene‒environment interaction effect of hypothalamic‒pituitary‒adrenal axis gene polymorphisms and job stress on the risk of sleep disturbances
Source: PeerJ. 2024 Mar 20;12:e17119. doi: 10.7717/peerj.17119 (PMC10960531; doi:10.7717/peerj.17119)
Supplement: Supplemental Information 2 — Loci chosen for hap-analysis: rs1360780, rs4713916, rs3777747, rs3800373, rs9296158, rs9470080. All alleles of the above SNPs were analyzed by haplotype, using the first allele of each SNP as the reference standard (results are shown in Table 4, e.g., OR (95% CI) = 1.00 for rs1360780-C allele), and alleles with a frequency of less than 0.03 in controls and cases were excluded. Global chi2 is 123.439682, while df = 10, P < 0.01. Loci chosen for hap-analysis: rs1360780, rs4713916, rs3777747, rs3800373, rs9296158, rs9470080. [file peerj-12-17119-s002.docx]

**Haplotype analysis of FKBP5 gene**

Haplotype analysis was performed on 6 SNPs of the FKBP5 between sleep disturbance group and non-sleep disturbance group subjects (those with haplotype frequencies <0.03 were excluded from the analysis), and 9 common haplotypes were established (Table S1). The results showed that there were significant differences in haplotypes between the sleep disturbance group and the non-sleep disturbance group. The C-A-G-A-G-C haplotype is associated with an increased risk of sleep disturbance. However, the C-A-A-A-A-C, C-G-G-A-G-C and C-G-G-A-G-T haplotypes were protective factors for sleep disturbances.

**Table S1 Haplotype analysis of FKBP5 gene**

| Haplotype | Sleep disturbance (F) | Non-sleep disturbance (F) | OR（95%CI） | *P-*value |
| --- | --- | --- | --- | --- |
| C-A-A-A-A-C | 30.96 (0.06) | 85.56 (0.11) | 0.57 (0.37-0.88) | **0.01** |
| C-A-G-A-G-C | 27.89 (0.05) | 5.01 (0.01) | 9.90 (3.80-25.85) | **<0.01** |
| C-G-A-A-A-C | 42.25 (0.08) | 62.31 (0.08) | 1.15 (0.76-1.73) | 0.51 |
| C-G-A-A-G-C | 44.64 (0.08) | 58.92 (0.07) | 1.30 (0.86-1.96) | 0.21 |
| C-G-G-A-G-C | 134.43 (0.25) | 308.63 (0.38) | 0.60 (0.46-0.77) | **<0.01** |
| C-G-G-A-G-T | 8.14 (0.02) | 34.38 (0.04) | 0.38 (0.18-0.83) | **0.01** |
| T-A-G-C-A-T | 17.22 (0.03) | 28.34 (0.04) | 1.02 (0.55-1.87) | 1.00 |
| T-G-A-C-G-T | 27.44 (0.05) | 54.00 (0.07) | 0.84 (0.52-1.35) | 0.47 |
| T-G-G-C-G-T | 40.63 (0.08) | 54.20 (0.07) | 1.28 (0.84-1.96) | 0.26 |

Note: Loci chosen for hap-analysis: rs1360780, rs4713916, rs3777747, rs3800373, rs9296158, rs9470080. All alleles of the above SNPs were analyzed by haplotype, using the first allele of each SNP as the reference standard (results are shown in Table 4, e.g., OR (95% CI) = 1.00 for rs1360780-C allele), and alleles with a frequency of less than 0.03 in controls and cases were excluded. Global chi2 is 123.439682, while df=10 , *P* **<** 0.01. Loci chosen for hap-analysis: rs1360780, rs4713916, rs3777747, rs3800373, rs9296158, rs9470080.
