# Supplementary material for: Gene‒environment interaction effect of hypothalamic‒pituitary‒adrenal axis gene polymorphisms and job stress on the risk of sleep disturbances
Source: PeerJ. 2024 Mar 20;12:e17119. doi: 10.7717/peerj.17119 (PMC10960531; doi:10.7717/peerj.17119)
Supplement: Supplemental Information 3 — Adjusted for gender, age, ethnicity, marital status, smoking status and alcohol status. The best interaction model was selected based on the balance test error of the 1/10 test sample, the accuracy of the cross-validation and P-value, suggest that rs1360780 ×rs947008 ×rs110402 is the best interaction model (Cross-Validation Consistency:10/10, P < 0.001). Statistically significant P value was denoted in bold. [file peerj-12-17119-s003.docx]

**Table S2 Best gene-gene interaction models, as identified by GMDR**

| Model | Training Accuracy (%) | Testing Accuracy (%) | Cross-Validation Consistency | P-value |
| --- | --- | --- | --- | --- |
|  |  |  |  |  |
| rs1360780 | 0.57 | 0.56 | 10/10 | **0.01** |
| rs1360780×rs110402 | 0.59 | 0.54 | 9/10 | 0.05 |
| rs1360780×rs947008×rs110402 | 0.61 | 0.57 | 10/10 | **<0.001** |

Note: Adjusted for gender, age, ethnicity, marital status, smoking status and alcohol status. The best interaction model was selected based on the balance test error of the 1/10 test sample, the accuracy of the cross-validation and P-value, suggest that rs1360780 ×rs947008 ×rs110402 is the best interaction model (Cross-Validation Consistency:10/10, *P*<0.001). Statistically significant P value was denoted in bold.
