# Supplementary material for: Gene‒environment interaction effect of hypothalamic‒pituitary‒adrenal axis gene polymorphisms and job stress on the risk of sleep disturbances
Source: PeerJ. 2024 Mar 20;12:e17119. doi: 10.7717/peerj.17119 (PMC10960531; doi:10.7717/peerj.17119)
Supplement: Supplemental Information 5 [file peerj-12-17119-s005.docx]

**Data-introduction**

Gender: male=1, female=2

Age: ≤30=1, 31-40=2, 41-50=3, ＞51=4

Ethnicity: Han=1, Minority=2

Marital status: Unmarried=1, Married=2, Divorced=3

Smoking status: Non-smoker=1, Smoker=2

Alcohol consumption: Non-drinker=1, Drinker=2

ERI group: ERI≤1=1, ERI>1=2

PSQI group: Normal=0, Sleep Disturbance=1

rs1360780a: CC=1, CT =2, TT =3

rs3800373a: AA=1, AC=2, CC=3

rs9470080a: CC=1, CT=2, TT=3

rs4713916a: GG=1, GA=2, AA=3

rs3777747a: AA=1, GA=2, GG=3

rs9296158a: GG=1, GA=2, AA=3

rs110402a: AA=1, GA=2, GG=3

rs2267715a: AA=1, GA=2, GG=3

rs41423247a: GG=1, GC=2, CC=3
