# Supplementary material for: Gene‒environment interaction effect of hypothalamic‒pituitary‒adrenal axis gene polymorphisms and job stress on the risk of sleep disturbances
Source: PeerJ. 2024 Mar 20;12:e17119. doi: 10.7717/peerj.17119 (PMC10960531; doi:10.7717/peerj.17119)
Supplement: Supplemental Information 6 [file peerj-12-17119-s006.doc]

STROBE Statement—Checklist of items that should be included in reports of ***cross-sectional studies***

|  | Item No | Recommendation |
| --- | --- | --- |
| **Title and abstract** | 1 | **(*a*) Title :** Gene‒environment interaction effect of HPA axis gene polymorphisms and job stress on the risk of sleep disturbances |
|  |  | **(*b*) Abstract:** Background. Studies have shown that long-term exposure to job stress may increase the risk of sleep disturbances and that hypothalamic‒pituitary‒adrenal (HPA) axis gene polymorphisms may play an important role in the psychopathological mechanism underlying sleep disturbances. ...These findings may be used to improve sleep disturbances in the future.” |
| Introduction | | |
| Background/rationale | 2 | **See Introduction paragraph 1-4. Example text Introduction, paragraph 4:** “However, there are still SNPs in the HPA axis that have not been fully investigated in these interactions. More importantly, most studies have limited their focus to the effect of a single gene-stress interaction on sleep quality, and few have examined multiple major genes regulating the HPA axis to determine the relationships among gene polymorphisms, job stress, and their interaction with sleep disturbances.” |
| Objectives | 3 | **Introduction, paragraph 5 for study aims:** we examined the independent and interactive effects of HPA axis gene polymorphisms and job stress on sleep quality among front-line railway workers in Fuzhou City, China. Our investigation focused on the interaction effect of genetic and environmental factors on sleep disturbances to provide new insights for improving sleep health. |
| Methods | | |
| Study design | 4 | **Methods, ‘Study design’ :** “The present study was conducted as part of an Occupational Health Study for Railway Workers (OHSRW) between October 2019 and May 2020. nclusion and exclusion criteria have been described in detail in a previous article (Wang et al., 2022). A set of self-report questionnaires was used to collect information on demographic characteristics, sleep disturbances and job stress. As a part of physical examinations, 5-mL fasting venous blood samples were collected from each subject at the workplace between 7:00 am and 9:00 am. ” |
| Setting | 5 | **Methods, ‘Setting’ :** “The present study was conducted as part of an Occupational Health Study for Railway Workers (OHSRW) between October 2019 and May 2020. ” |
| Participants | 6 | **(*a*) See Methods, ‘Subjects’. Example text :** “The present study was conducted as part of an Occupational Health Study for Railway Workers (OHSRW) between October 2019 and May 2020. Inclusion and exclusion criteria have been described in detail in a previous article (Wang et al., 2022).” |
| Variables | 7 | **N/A** |
| Data sources/ measurement | 8* | **Methods, paragraphs 2-4. Example text :** “Job stress: The Effort-Reward Imbalance (ERI) scale was used to evaluate job stress, which is based on Siegrist's ERI model (Siegrist and Li 2017)...”, Sleep disturbances: The Pittsburgh Sleep Quality Index (PSQI) was used to assess the sleep quality of the subjects (Buysse et al., 1989)...”, “DNA Extraction and Genotyping: After a 12-hour fast, venous blood samples were collected from all participants using EDTA-containing tubes...” |
| Bias | 9 | **See Methods paragraphs 5 , ‘Confounding Factors’. Example text :** “  It has been demonstrated that some demographic, socioeconomic and lifestyle factors are related to sleep disturbances; thus, they may influence the results of any interaction between sleep disturbances and job stress or HPA axis gene polymorphisms (Wakasugi et al., 2014). The variables we included as confounders have been described in previous articles (Wang, Zhao et al., 2022). In particular, smoking and drinking alcohol were considered potential confounding lifestyle factors.” |
| Study size | 10 | **To identify the study population - Methods ‘Subjects’: Example text :** “In this cross-sectional study, a total of 690 participants were enrolled, of whom 19 were excluded due to insufficient information or missing blood samples. Ultimately, 671 (males/females =363/308) railway front-line workers were included in the final analysis. ” |
| Quantitative variables | 11 | **Methods ‘paragraphs 2-4’ and ‘Confounding Factors’. Example text :** “ERI scores>1 indicate an imbalance between effort and reward, which is considered to reflect job stress (Choi et al., 2014).” Subjects with a global score higher than 5 were classified as experiencing sleep disturbance (Liu et al., 2021).”  **Example text, ‘Confounding Factors’ :** “It has been demonstrated that some demographic, socioeconomic and lifestyle factors are related to sleep disturbances.” |
| Statistical methods | 12 | **(*a*) Methods ‘Statistical Analysis’. Example text :** “Statistical analyses were carried out using SPSS version 26.0 (SPSS Inc., Chicago, IL, USA). ERI and PSQI scores are presented as the mean ± standard deviation (SD). Demographic data between two groups were compared using the chi-squared test for categorical variables. The Hardy-Weinberg equilibrium (HWE) for the HPA axis gene polymorphisms was tested using a chi-squared goodness-of-fit test. Pearson correlation analysis was used to assess the correlations of job stress with sleep disturbances and its dimension scores. After adjusting for sex, age, ethnicity, marital status, smoking status and drinking status as covariates, odds ratios (ORs) and 95% confidence intervals (Levante et al., ) were determined for the association of genotypes and job stress with the risk of sleep disturbances by logistic regression. Bonferroni correction was applied to account for multiple comparisons. Furthermore, GMDR (http// sourceforge.ne/projects/gmdr/) was used to identify the best HPA axis gene × job stress combination (Xu et al., 2016). We conducted a 10-fold cross-validation (CV) to avoid unstable results and obtained a robust averaged result. We also conducted locus and haplotype analysis for haplotypes associated with sleep disturbances using SHEsis (http://analysis.bio-x.cn). ” |
|  |  | **(*b***)**Methods ‘Statistical Analysis’. Example text :** “Furthermore, GMDR (http// sourceforge.ne/projects/gmdr/) was used to identify the best HPA axis gene × job stress combination (Xu et al., 2016). We conducted a 10-fold cross-validation (CV) to avoid unstable results and obtained a robust averaged result.” |
|  |  | **(*c*) N/A** |
|  |  | **(*d*) N/A** |
|  |  | **(*e*) N/A** |
| Results | | |
| Participants | 13* | **(*a*) Methods ‘Subjects’ and Results ‘Demographic characteristics of the subjects’ : Methods ‘Subjects’ , Example text :** “In this cross-sectional study, a total of 690 participants were enrolled, of whom 19 were excluded due to insufficient information or missing blood samples. Ultimately, 671 (males/females =363/308) railway front-line workers were included in the final analysis. ”  **Results ‘Demographic characteristics of the subjects’ , Example text :** “A total of 671 subjects were included in this study, including 269 with sleep disturbances and 402 without sleep disturbances. ” |
|  |  | **(b)N/A** |
|  |  | **(c) N/A** |
| Descriptive data | 14* | **(a) Results ‘Demographic characteristics of the subjects’ , Example text :** “A total of 671 subjects were included in this study, including 269 with sleep disturbances and 402 without sleep disturbances. The incidence of sleep disturbances was 40.09%. We found no significant differences in sex, age, ethnicity, marital status, smoking status or drinking status between the two groups (P>0.05). In addition, there was a significant difference in the distribution of job stress between the two groups (P<0.01).” |
|  |  | **(b) N/A** |
| Outcome data | 15* | **See Table 2. Example text :**“The general demographic characteristics of the sleep-disturbance group and nonsleep-disturbance group are summarized in Table 2.” |
| Main results | 16 | 1. **Provided in Results ‘paragraphs 2-4’ and Table 3-5,Table S1.**   **Results paragraph 2 ‘Correlation between job stress and sleep disturbances’:**“Table 3 shows the correlations among the ERI scores, PSQI scores, and all dimensions of sleep disturbances.”  **Results paragraph 3 ‘Associations of 9 HPA axis SNPs with sleep disturbances’:**“The associations of 9 SNPs in the HPA axis with sleep disturbances are presented in Table 4. ”  **Results paragraph 4 ‘Effect of the gene–environment interaction on sleep disturbance’:**“The best gene‒environment interaction models were determined by GMDR analysis (Table 5). ” |
|  |  | **(*b*)N/A** |
|  |  | **(*c*)N/A** |
| Other analyses | 17 | **Results paragraph 4 ‘Effect of the gene–environment interaction on sleep disturbance’,Table 5 and Table S1.**  **Example text :** “The best gene‒environment interaction models were determined by GMDR analysis (Table 5).These models showed a significant effect of the interaction between HPA axis genes, job stress on sleep disturbance. ” |
| Discussion | | |
| Key results | 18 | **Discussion paragraph 1** |
| Limitations | 19 | **Discussion paragraph 5, Example text :** “However, this research still has some limitations that can be addressed in future studies. First, the evaluation of sleep disturbances was entirely based on the PSQI, which is a subjective questionnaire, and it is easy to produce false positive results, which may have affected the accuracy of results. ” |
| Interpretation | 20 | **Discussion paragraph 2-5** |
| Generalisability | 21 | **See ‘Conclusions’** |
| Other information | | |
| Funding | 22 | This study was supported by the Fujian Medical University’s Research Foundation for Talented Scholars (grant number XRCZX2018011), Fuzhou Science and Technology Project (grant number 2022-S-033). |

*Give information separately for exposed and unexposed groups.

**Note:** An Explanation and Elaboration article discusses each checklist item and gives methodological background and published examples of transparent reporting. The STROBE checklist is best used in conjunction with this article (freely available on the Web sites of PLoS Medicine at http://www.plosmedicine.org/, Annals of Internal Medicine at http://www.annals.org/, and Epidemiology at http://www.epidem.com/). Information on the STROBE Initiative is available at www.strobe-statement.org.
