# Supplementary material for: Gene‒environment interaction effect of hypothalamic‒pituitary‒adrenal axis gene polymorphisms and job stress on the risk of sleep disturbances
Source: PeerJ. 2024 Mar 20;12:e17119. doi: 10.7717/peerj.17119 (PMC10960531; doi:10.7717/peerj.17119)
Supplement: Supplemental Information 8 [file peerj-12-17119-s008.docx]

1.OLIGO_Name Start Length Tm Gc com 3'com Rep seq

rs41423247F 217 22 65.37 59.09 3 1 0 GGGGATGAGGTTACGGGGTAGA

rs41423247R 548 22 65.07 50.00 3 2 0 TGCTCACAGGGTTCTTGCCATA

TITLE:rs41423247 NUM:1 SNP:0 Strand:1

rs41423247FG:TTCCGCGTTCGGACTGATAT GTTGACACCAATTCCTCTCTTAAAGAGAGTG 66.08

rs41423247FC:TACGGTTATTCGGGCTCCTGT GTTGACACCAATTCCTCTCTTAAAGAGAGTC 65.41

rs41423247FP:ATCAGCAGACATAACTTGTCTACTTTATGGC TTTTTTTTT 64.80

2.OLIGO_Name Start Length Tm Gc com 3'com Rep seq

rs3800373F 361 22 65.35 54.55 5 1 0 GGCATGGGAAGCTGTCTTCAAC

rs3800373R 767 24 66.66 54.2 7 0 CCAGCATTGCTACTGCTCAGCTTC

TITLE:rs3800373 NUM:1 SNP:0 Strand:2

rs3800373FC:TCTCTCGGGTCAATTCGTCCTT CCTAGTGTAGAAGAGCAACTATTTATTTGTCATCC 65.79

rs3800373FA:TGTTCGTGGGCCGGATTAGT CCTAGTGTAGAAGAGCAACTATTTATTTGTCACCA 65.42

rs3800373FP:CTACAGATTTTGTTTTTAAAAATTAAGCTTAGTACT TTT 60.88

3.OLIGO_Name Start Length Tm Gc com 3'com Rep seq

rs9296158F 343 28 65.01 42.9 4 2 0 CACTCGTTCTGTTATACTCATTCCATGC

rs9296158R 575 22 65.64 59.09 6 2 0 AGGCCTGGGCTAGGGGTAATTC

TITLE:rs9296158 NUM:1 SNP:0 Strand:2

rs9296158RG:TTCCGCGTTCGGACTGATAT GGAATTAGATTTGAAGCTGGTTTCTGGACTAGAC 67.17

rs9296158RA:TACGGTTATTCGGGCTCCTGT GGAATTAGATTTGAAGCTGGTTTCTGGACTAGAT 66.77

rs9296158RP:TGATATTGAGAGTGATATTACCAGGTCATAGA TTTTTTTT 63.29

4.OLIGO_Name Start Length Tm Gc com 3'com Rep seq

rs3777747F 327 22 65.04 54.55 4 1 0 CCGCCTAAGCCTGTTGAGAAGA

rs3777747R 601 22 65.21 54.55 4 0 0 TCCAGTTGTTGGCGTACCTCCT

TITLE:rs3777747 NUM:1 SNP:0 Strand:1

rs3777747FG:TTCCGCGTTCGGACTGATAT CTCTTACATTCCTCTCCTTTCCATCG 66.44

rs3777747FA:TACGGTTATTCGGGCTCCTGT CTCTTACATTCCTCTCCTTTCCACCA 65.10

rs3777747FP:MTATTATTGATCTCTTCTTCTTCTTTTGAAAATCT TTTTTTT 63.01

5.OLIGO_Name Start Length Tm Gc com 3'com Rep seq

rs1360780F 208 22 64.76 50.00 4 2 0 GGCATGGGCACTCTGAAAAGAT

rs1360780R 542 24 64.77 50.00 4 2 0 TCTCTTGTGCCAGCAGTAGCAAGT

TITLE:rs1360780 NUM:1 SNP:0 Strand:1

rs1360780FC:TCTCTCGGGTCAATTCGTCCTT CACAGAAGGCTTTCACATAAGCAAAGTGAC 65.98

rs1360780FT:TGTTCGTGGGCCGGATTAGT CACAGAAGGCTTTCACATAAGCAAAGTGAT 65.53

rs1360780FP:ACAAAACAAAAATTCTTACTTGCTACTGCTG TTTTTTT 64.74

6.OLIGO_Name Start Length Tm Gc com 3'com Rep seq

rs9470080F 423 26 64.06 34.62 5 3 0 TCTTTTCCAGGCTATGAATTGACAAA

rs9470080R 653 22 64.97 45.45 6 0 0 TGTGTCCAGCCATGTGCTTTTT

TITLE:rs9470080 NUM:1 SNP:0 Strand:1

rs9470080FC:TCTCTCGGGTCAATTCGTCCTT CAGTTTCATAATTACCATTTGTCCAAAGTCTAC 66.81

rs9470080FT:TGTTCGTGGGCCGGATTAGT CAGTTTCATAATTACCATTTGTCCAAAGTCCAT 66.39

rs9470080FP:CTCTGAGCTAAAACACAATGTTTTTTATGTT TTTTTTT 63.07

7.OLIGO_Name Start Length Tm Gc com 3'com Rep seq

rs4713916F 461 22 65.29 54.5 4 2 0 TGGCAACCCTAACCTCTCTGGA

rs4713916R 654 24 64.28 50.00 6 0 0 TGTAGGTTCGGGGTACATGTGAAG

TITLE:rs4713916 NUM:1 SNP:0 Strand:2

rs4713916RG:TTCCGCGTTCGGACTGATAT CGAGATAGTGAGGAGTTATTGGACCAACAC 66.95

rs4713916RA:TACGGTTATTCGGGCTCCTGT CGAGATAGTGAGGAGTTATTGGACCAACAT 66.48

rs4713916RP:AGAGGAAAATGTAGGAGTCCAGAGAGG TTTTTTTT 64.46

8.OLIGO_Name Start Length Tm Gc com 3'com Rep seq

rs2267715F 335 22 64.67 54.55 8 0 0 TCTCTCCCAGCAGGGAAGTTGT

rs2267715R 601 22 64.75 59.09 3 1 0 CTGGAGGGAGTGGGGGTAAACT

TITLE:rs2267715 NUM:1 SNP:0 Strand:2

rs2267715RG:TCTCTCGGGTCAATTCGTCCTT GTGCAAGCCTCAGGAGGAACC 67.74

rs2267715RA:TGTTCGTGGGCCGGATTAGT GTGCAAGCCTCAGGAGGAGCT 65.30

rs2267715RP:CAGATAGACCCTGCCCTCTGG TTTTTTTT 63.32

9.OLIGO_Name Start Length Tm Gc com 3'com Rep seq

rs110402F 278 22 65.01 54.55 6 0 0 AGATCAGCGGATGGTGAAGAGG

rs110402R 590 22 64.53 59.09 7 1 0 CTTGGCTGCCTAGAACCCTGAC

TITLE:rs110402 NUM:1 SNP:0 Strand:1

rs110402FG:TCTCTCGGGTCAATTCGTCCTT GAATTTTAAGAAGCATTTTTCTTTGCATATCG 66.19

rs110402FA:TGTTCGTGGGCCGGATTAGT GAATTTTAAGAAGCATTTTTCTTTGCATACCA 65.14

rs110402FC:TACGGTTATTCGGGCTCCTGT GAATTTTAAGAAGCATTTTTCTTTGCATAACC 65.14

rs110402FP:CAACACCAGTCCTCTGTGTTTAGAAAA TTTTTTTTTTTTTTTTTTTTTTTTTT 63.81
